# Living on the Edges: Spatial Niche Occupation of Asian Citrus Psyllid, *Diaphorina citri* Kuwayama (Hemiptera: Liviidae), in Citrus Groves

**DOI:** 10.1371/journal.pone.0131917

**Published:** 2015-07-20

**Authors:** Mamoudou Sétamou, David W. Bartels

**Affiliations:** 1 Department of Agriculture, Agribusiness and Environmental Sciences, Texas A&M University-Kingsville Citrus Center, Weslaco, Texas, United States of America; 2 Center for Plant Health Science Technology, Plant Protection Quarantine—Mission Laboratory USDA-APHIS, Mission, Texas, United States of America; United States Department of Agriculture, UNITED STATES

## Abstract

The spatial niche occupation of the Asian citrus psyllid, *Diaphorina citri* Kuwayama, 1908, was evaluated to determine its field colonization and food resource exploitation strategies in citrus groves. Mature grapefruit and sweet orange groves were surveyed as part of an area-wide program in 2009–2010 to determine *D*. *citri* population densities and between-tree distribution. In both cultivars, significantly more psyllids were found on perimeter trees throughout the study period suggesting a strong edge effect in *D*. *citri* distribution in the groves. *D*. *citri* densities and infestation levels gradually declined from the edge to the center of grove. Higher numbers of *D*. *citri* were recorded on trees located on the east and south sides of the groves than those on the west and north sides. Citrus groves located at the outer edge of the study with at least one side non-surrounded to other citrus groves harbored significantly more *D*. *citri* than groves located within the block cluster and entirely surrounded by other groves. In detailed field studies during 2012, infestation of *D*. *citri* started from border trees in the grove where possibly one generation is completed before inner trees become infested. In addition, psyllid densities decreased significantly with increasing distance from the grove edge. Using the selection index, *D citri* exhibited a strong niche occupation preference for border trees.

## Introduction

The Asian citrus psyllid, *Diaphorina citri* Kuwayama, 1908 (Hemiptera: Liviidae), has emerged as a major pest in the United States in recent years. Since its first detection in Florida in 1998 [1], *D*. *citri* has spread across all citrus producing states in the U.S. [2,3]. Sprawling citrus groves intermingled with abundant dooryard citrus trees that remain largely unmanaged in all commercial citrus producing states, have probably favored the rapid spread of the psyllid. *Diaphorina citri* is economically important because it is a known vector of *Candidatus* Liberibacter asiaticus (CLas), the putative bacterial causal agent of citrus greening disease, also called Huanglongbing (HLB) [4,5]. Presently, there is no cure for HLB and infected citrus trees gradually decline and produce unmarketable fruit, and eventually die. Since *D*. *citri* is the primary means of spread of CLas between trees, vector control to prevent the spread of the disease is one of the recommended strategies to mitigate HLB worldwide [6].

Development of effective management strategies for insect pests requires an understanding of their spatial distribution patterns within the agricultural landscape [7]. Despite the uniformity of trees in citrus groves, all development stages of *D*. *citri* exhibit an aggregated behavior on flush shoots, where egg laying and immature development occur [4]. Adult psyllids are known to move frequently between groves [8], but very little research has investigated the spatial distribution between trees within groves and between groves at the landscape level, and filling this gap will help in the development of more effective psyllid management programs. Higher than expected numbers of HLB-infected trees were recorded at the edge of citrus groves in China, Brazil, and Florida, and this observation was attributed to possible psyllid accumulation at the interface or edges of citrus groves [9,10]. In addition to these anecdotal reports on the edge effect in *D*. *citri* migration and spatial distribution in citrus groves, other reports have also noted higher psyllid densities and citrus flush shoot infestation levels on the southern and eastern sides of groves [4]. Similarly, edge effects were also recorded in the field distribution of potato zebra chip, a disease caused by a *Candidatus* Liberibacter solanacearum and its psyllid vector *Bactericera cockerelli* (Sulc.) (Hemiptera: Psyllidae) [11].

In agricultural landscapes, insect pest population densities can change from the edge to the interior of a habitat, resulting in edge effects [12]. Edge effects in insect spatial distribution may be due to several factors including insect behavior, host plant distribution, availability of oviposition and feeding sites, distribution and densities of natural enemies, windbreaks, and microclimates [12–14]. Edge effects are processes resulting from an abrupt transition between two adjacent ecosystems or patches with differing qualities [15,16]. Thus, the edge of a citrus grove may be defined as an interface between a dense citrus planting and an area void of citrus [10]. Boundaries of individual citrus blocks can be delineated by roads, irrigation and drainage canals, ponds, another citrus block with distinctive vegetation, any vegetation other than citrus, or any different geographic feature.

The spatial distribution and niche occupation of *D*. *citri* in citrus are poorly understood. Elucidating spatial patterns of this economically important disease vector is important for the development of an effective management program. Currently, psyllid control in commercial groves is mostly done via application of insecticides to the whole blocks, without consideration of psyllid location in the block or how psyllid colonization starts within the grove and progresses through the season. In this study, we investigated the spatial distribution and niche occupation of all *D*. *citri* life stages within citrus blocks, and between blocks on an area-wide basis. We also evaluated the pattern of individual grove colonization by adult psyllids. The main objective of this study was to determine whether *D*. *citri* exhibited an edge effect and niche occupation preference, and to estimate the rate of change of psyllid density from the edge to the interior of the grove.

## Materials and Methods

### Area-wide surveys

Surveys were conducted in 2009–2010 as part of an area-wide program to collect data on psyllid infestation levels and population densities in mature blocks of commercial citrus covering ∼583 ha (1,457 acres) planted in several cultivars of two citrus species (*Citrus sinensis* (L.) Osbeck [sweet orange] and *C*. *×paradisi* Macfad. [grapefruit]) in the Lower Rio Grande Valley (LRGV) of South Texas (See S1 **Fig**). The groves are privately owned by two large citrus growing companies (Rio Queen Citrus and Healds Valley) who granted full permission for accessing the groves and conducting these collaborative surveys, which did not involve any endangered or protected species. Only the target insect vector *D*. *citri* was sampled during the surveys. In the study area there were 107 grapefruit blocks covering 415 ha while the 69 sweet orange blocks covered 168 ha, reflecting the species composition of commercial citrus in the LRGV. The study area was divided into grid cells of 1,600 m^2^ each, and a sampling site was selected at grid corners along the perimeter of a citrus block, independent of the cultivar. Sampling site selection was made such that the entire study area was uniformly covered. A total of 99 and 54 sampling sites were selected for grapefruit and sweet orange, respectively. The location of the citrus grove being sampled relative to the entire study area was recorded as outer or inner blocks when the block was either at the edge or entirely surrounded by other groves in the study area, respectively. At each sampling site, three sentinel trees were surveyed. The first tree at a corner along the perimeter of the grove labeled as “perimeter tree”, the second tree was the adjacent one on the next row inside the grove termed “adjacent tree” and the third tree was a randomly selected tree row within the grove along a diagonal through the perimeter tree from the 8^th^ to the 10^th^ row inside the grove labelled as “interior tree”. Given the diversity of planting densities of groves, the mean distance of the adjacent and interior tree relative to the perimeter tree was calculated as 7 m and 40 m, respectively. All surveyed groves belonged to two large citrus companies that use standard grove care practices, including conventional pest control programs.

At each sampling site, densities of all *D*. *citri* developmental stages were monitored on the three sentinel trees (perimeter, adjacent and interior) every two weeks using visual observation. The canopy of each tree was divided into four quadrants and five flush shoots were randomly selected per quadrant for a total of 20 flush shoots per tree. The developmental stage of flush shoots was first recorded following a scale developed by Arredondo [17]. Based on the presence of young flush shoots, the sampled grove was recorded as ‘flushing’. Care was taken to sample the most juvenile flush stages when present. Selected flush shoots were carefully examined for the presence of any *D*. *citri* developmental stage. Numbers of adults were recorded first, and then using a 10 x hand lens, the numbers of eggs and nymphs were counted *in situ* and recorded per flush. However immature *D*. *citri* were only recorded on young shoots-developmental stages 1 to 3- as reported by Arredondo [17]. Percent flush shoot infestation level was determined as the ratio of flush shoots infested with any *D*. *citri* developmental stage over the total number of shoots sampled per tree for each sampling period.

### *D*. *citri* field colonization and dispersal strategies in field study

A detailed field study to evaluate the field colonization and niche occupation patterns of *D*. *citri* adults was conducted in two adjacent ‘Rio Red’ grapefruit groves (See S2 Fig) at the Texas A&M University-Kingsville Citrus Center, Weslaco, Texas, in 2012. One of the blocks was a mature block of 12 year old trees, while the second one was a young block of 4 year- old trees. Both blocks were sprayed twice during the dormant period (November to early February) of citrus trees in South Texas when no new flush shoots and immature stages of *D*. *citri* were present, and did not received any insecticide application for the reminder of the trial. Both sprays were done early in the morning between 06:00 and 09:00 using a tractor mounted airblast sprayer delivering about 1,870 L/ha of total spray volume. The first spray application on 4 January 2012 was done with fenpropathrin at 0.34 kg/ha (Danitol 2.4 EC, Valent USA Corporation, Walnut Creek, CA) while the second spray was applied on 25 January 2012 with the neonicotinoid imidacloprid at 0.28 kg a.i./ha (Provado 1.6 F, Bayer CropScience, Research Triangle Park, NC).

Starting from one week after the second spray application, *D*. *citri* adult populations were monitored in the two groves using lime-green sticky card known as ACP-Trap (Alphascents Inc., West Linn, OR) from February to November, corresponding to an average citrus crop season in Texas. Five sampling transects were established across all rows in each grove. The first and fifth transects were along the first trees at both row-ends of the grove, while the three middle transects were equidistantly placed from the row-end transects. Because the young citrus grove has a trapezoidal shape, transect lines were established along the shortest base (See S2 Fig). A trap was deployed on all trees across a transect line, and attached at ≈ 1.5 m above ground directly on tree canopy with a tie such that a sticky surface was facing the outside. Using this sampling scheme, traps could be grouped into perimeter, adjacent and interior trees (see S2 Fig). Traps were initially replaced every two weeks until adult psyllids were detected at each trapping location and monthly thereafter. Recovered traps were brought to the laboratory, examined under a stereomicroscope, and the number of *D*. *citri* adults tallied per trap.

### Data analysis

#### Area-wide survey data

A repeated measure analysis via Proc Mixed of SAS [18] was used to determine the effect of citrus species (grapefruit and sweet orange), grove location in the study area (outer or inner), tree position in the grove (perimeter, adjacent and interior), sampling time and their interactions on *D*. *citri* densities, and flush shoot infestation level. The variables for citrus species and grove location were fixed factors, while tree position was considered a random factor and sampling time was a continuous random variable. Whenever the effect of a factor or variable was significant, least squared means were compared using Tukey test with the LSMeans procedure of SAS. Numbers of *D*.*citri* developmental stages per flush shoot were log(x+1)-transformed and percent flush shoot infestation levels were arcsine-transformed before analysis [19].

Because *D*. *citri* densities and flush shoot infestation levels significantly decreased from the perimeter to the interior tree, the rate of decline was assessed using an exponential decay function (*y* = a*exp [*-bx*]). Additionally, simple linear regression analysis was performed to evaluate the relationships between *D*. *citri* adult densities at various tree positions in a grove. All analyses were performed using SAS version 9.2 [18].

Mean cumulative numbers of *D*. *citri* per flush shoot were calculated for each life stage over sampling periods. The non-linear regression function of SigmaPlot (Version 12) was used to fit these cumulative data to a three-parametric sigmoidal function.

#### Field study data

Similar to the area-wide survey data, a repeated measure analysis was used to determine the effect of tree age (young and mature), tree position (perimeter, adjacent and interior), sampling time, and their interactions on *D*. *citri* adult densities. Simple linear regression analysis was also performed to evaluate the relationships between *D*. *citri* adult densities at various tree positions in a grove. All analyses were performed using SAS version 9.2 [18].

Cumulative numbers of *D*.*citri* adults caught on sticky traps were calculated over for each trapping position over the sampling period for both the young and mature grapefruit groves, and these data were fit to a sigmoidal function as previously described.

#### Niche occupation of D. citri in citrus during area-wide surveys and field study

To better understand the field distribution behavior of *D*. *citri* in relatively uniform tree stand in groves, its niche occupation parameters were calculated. Using the mean cumulative number of adult psyllids recorded on flush shoots during the area-wide study over the sampling periods for each tree position (perimeter, adjacent or interior) and adult psyllids caught per trap in the field study, the niche occupation preference of *D citri* was determined using the forage ratio or selection index [20,21] defined as:

$$w_{i}=o_{i}/p_{i}$$

Where *w*_i_ = selection index of *D*. *citri*; *o*_*i*_ = proportion or percentage of *D*. *citri* adults collected per tree position and *p*_*i*_ = proportion or percentage of trees available per position in the grove. Selection indices above 1.0 indicate habitat preference; values less than 1.0 indicate avoidance. However, the selection index as defined varies from 0 to ∞, which is a nuisance; consequently, Manly et al. [21] suggested presenting the selection index as standardized ratios that add up to 1.0 for all resource types:

$$B_{i}=w_{i}/\overset{n}{\underset{i=1}{\sum }}w_{i}$$

where *B*_*i*_ = standardized selection index for *D*. *citri* and *w*_*i*_ = forage ratio or selection index for *D*. *citri*. Standardized indices of selection equal to 1/number of resources available indicate no preference, whereas values below this indicate relative avoidance, and values above indicate relative preference [21].

For the 2009–2010 area-wide surveys, mean grove size and mean proportions of perimeter, adjacent and interior trees were derived for both the grapefruit and sweet orange blocks and used to determine *D*. *citri* niche occupation for each host plant species. The total number of trees in the grove and the proportion of trees within each position (perimeter, adjacent and interior) were tallied for each of the two groves in the 2012 field study and used to calculate the corresponding indices of *D*. *citri* habitat preference. A log-likelihood ratio test (*G*-test) was used to test the null hypothesis of random selection of habitat by *D*. *citri* in a citrus grove [21]. Whenever a significant *G*-test was obtained, the confidence intervals of the standardized selection indices were compared to discriminate between the three niche occupation indices of perimeter, adjacent and interior trees. All selection indices were computed using the niche occupation software of Krebs [22].

#### Geostatistical analysis of field study data

Trapping locations (n = 105) for the mature and young orchards were heads-up digitized into ArcGIS 10.2 (ESRI, Redlands, CA) using 6” natural color aerial imagery (2011) from the International Boundary Waters Commission. The imagery was in the North American Datum (NAD83) and projected to UTM zone 14 North. Monthly trapping data from May through October was joined to each trap location. Monthly data set was characterized to determine the spatial structure and meet the assumption of asymptotic spatial dependence prior to developing contour maps [23]. Various kriging methods were evaluated prior to settling on Empirical Bayesian Kriging (EBK). EBK automatically calculates the model parameters through a process of subsetting and simulations. EBK also accounts for the error introduced by estimating the underlying semivariogram through multiple simulations. Cross-validation of all models was used to assess the accuracy of the predicted surfaces. EBK model parameters provided a root-mean-square standardized error close to 1 (0.969–1.098) for all 6 months when *D*. *citri* were collected in groves.

## Results

### Spatio-temporal distribution of *D*. *citri* in citrus groves during area-wide surveys

**Fig 1:**
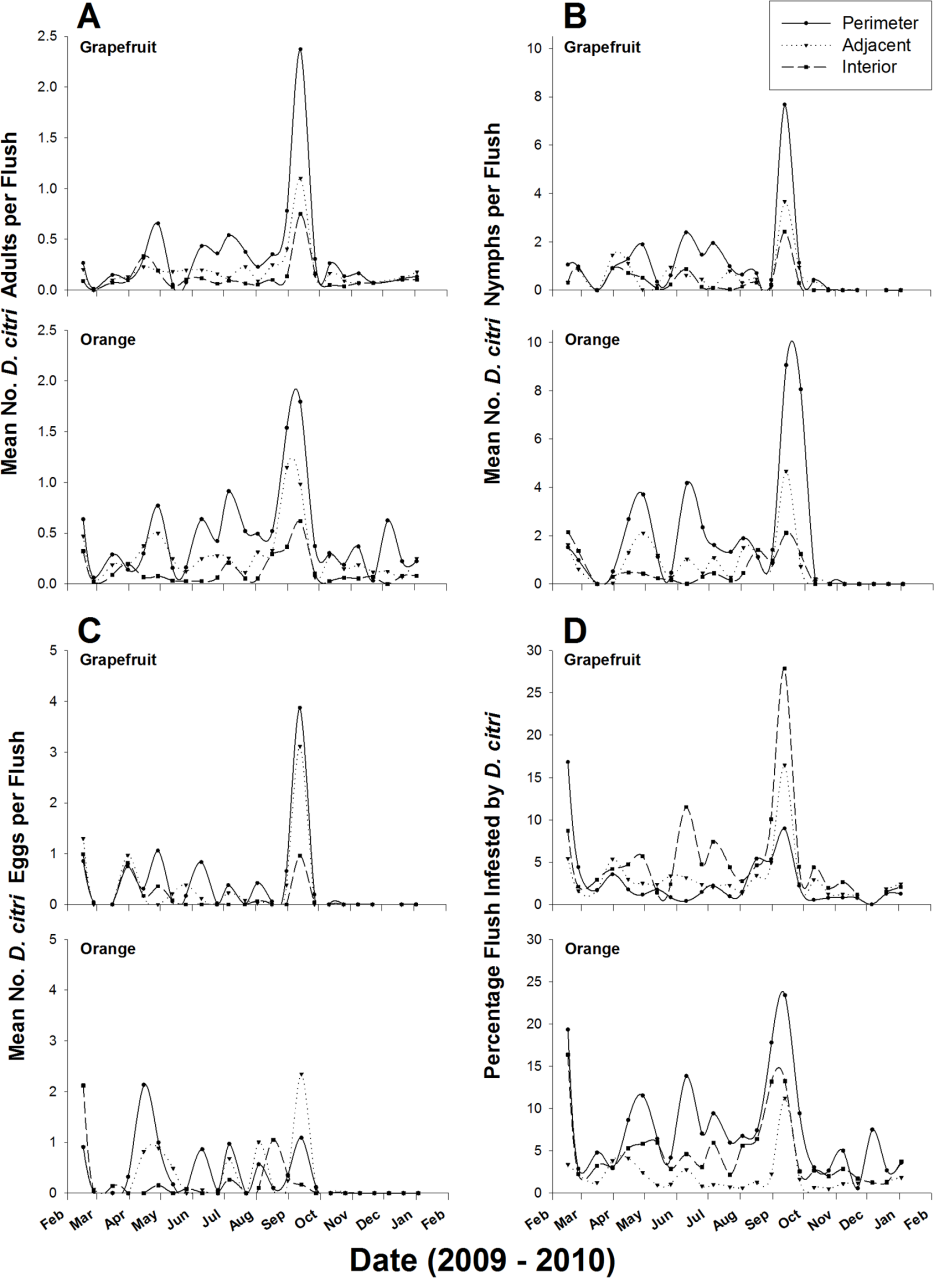
*Diaphorina citri* flush shoot infestation levels and population fluctuations on citrus trees depending on their position in the grove during area-wide surveys (2009–2010).

Densities of all *D*. *citri* life stages and flush shoot infestation levels on citrus greatly varied with sampling time (*F* > 32.46; df = 1, 35,000, *P* < 0.0001; Table 1, Fig 1) and the phenology of citrus trees. Four major flush cycles were observed in the groves during the survey period: February-March, May-June, July-August and September-October. Psyllid populations increased during flush cycles when new flush shoots were produced by trees and declined thereafter. There was a gradual increase in flush shoot infestation levels by *D*. *citri* in citrus groves from February to September-October when peak infestation levels were observed in Texas, but lower infestation levels were observed during the flush cycle of July-August before dramatically increasing during the last flush of the fall flush cycle in September-October (Fig 1). Despite this trend of gradual increase in flush shoot infestation levels with time during flush cycles, *D*. *citri* densities on flush shoots did not show a pattern of gradual increase, with the exception of adult numbers in sweet orange groves. Egg densities recorded on flush shoots during the summer flush cycles in June-August were significantly lower than those observed during any other flush cycle, while densities of nymphs were lowest during the summer flush cycle (July-August) in both citrus cultivars. The highest densities of all *D*. *citri* life stages were observed during the fall flush cycle in September-October (Fig 1).

**Table 1:** Mixed model analysis of variance showing *F*-values^1^ of major parameters affecting *D*. *citri* densities and infestation levels in mature citrus groves in Texas (2009–2010).

Effect	Numerator DF	*#* eggs/ flush shoot	*#* nymphs /flush shoot	*#* adults /flush shoot	% flush shoots infested
Host Plant	1	1.55^ns^	69.59**	76.34**	158.08**
Grove location	1	18.09**	21.16**	36.20**	41.78**
Tree position in grove	2	14.73**	99.45**	179.72**	221.72**
Sampling period	23	32.46**	69.28**	75.31**	85.03**

Error df for all analysis = 35,000.

^1^ ns = non-significant (P>0.05).

** = highly significant (P < 0.01).

With the exception of the numbers of eggs per flush shoot (*F* = 1.55; df = 1, 35,000; *P* = 0.22), densities of *D*. *citri* adults (*F* = 76.34; df = 1, 35,000; P < 0.0001) and nymphs (*F* = 69.59; df = 1, 35000; *P* < 0.0001) significantly varied with the host plant species (Table 1). Sweet orange flush shoots harbored significantly more psyllids nymphs and adults and were more infested (*F* > 69.59; df = 1, 35000; *P* < 0.0001) than grapefruit shoots. The least squared mean numbers (± SE) of *D*. *citri* nymphs (1.03 ± 0.06) and adults (0.32 ± 0.01) per flush shoot on sweet orange were respectively 1.7 and 1.4-fold higher than those recorded on grapefruit (0.62 ± 0.05 for nymphs and 0.23 ± 0.01 for adults), while *D*. *citri* flush shoot infestation level on sweet orange (5.2%) was 1.5-fold more infested than the level recorded on grapefruit (3.5%).

**Fig 2:**
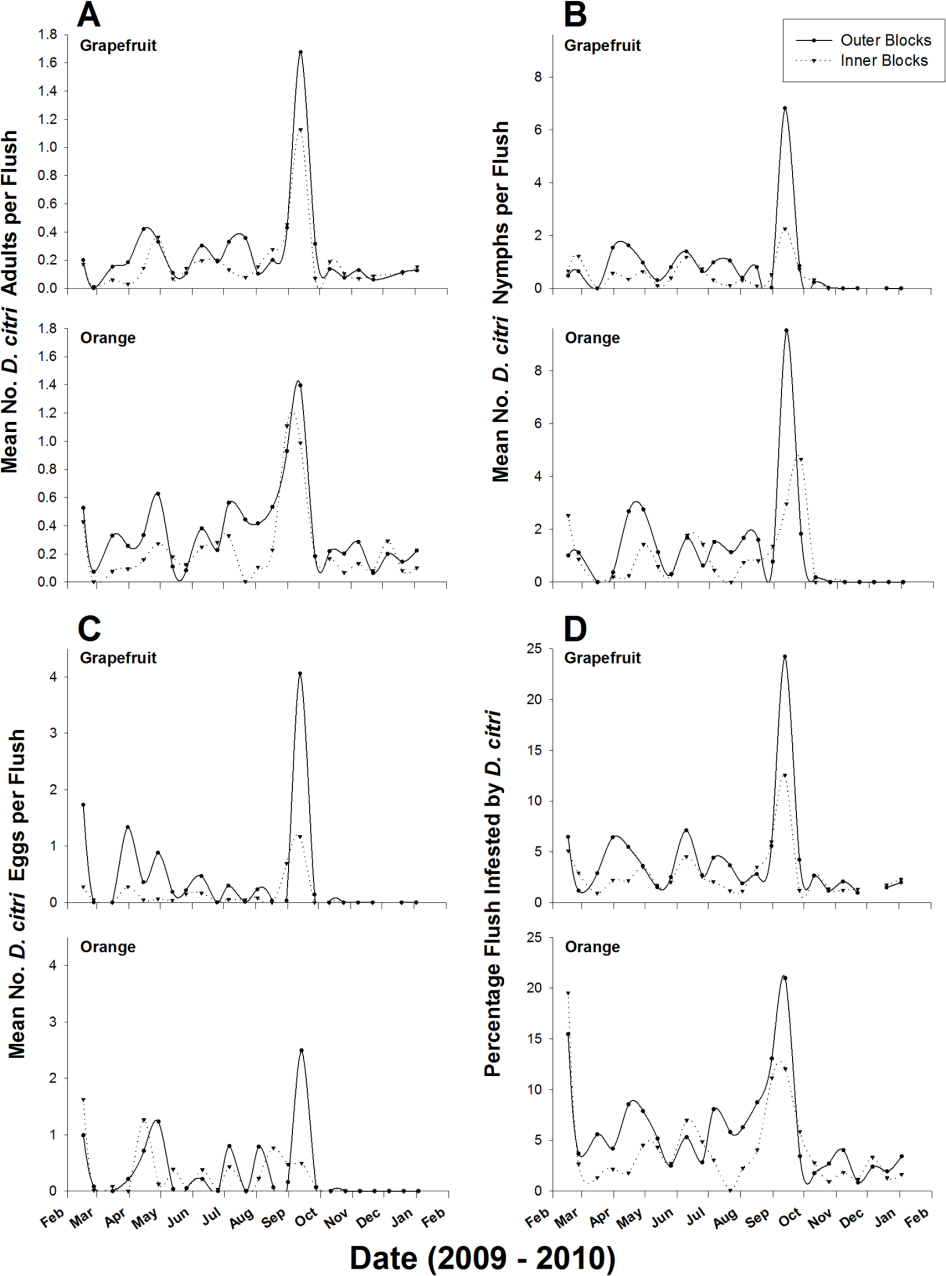
Comparative *D*. *citri* flush infestation levels and densities on trees of inner and outer groves in the area-wide study sites (2009–2010).

The location of grove also significantly affected *D*. *citri* infestation and densities recorded on trees (Table 1). Groves located at the outer edge of the study area harbored significantly more psyllids and had more infested flush shoots than inner groves surrounded by other citrus blocks (Fig 2). Regardless of host plant species and time of the year, densities of *D*. *citri* adults, nymphs and eggs were respectively 1.4, 1.5 and 2.0-fold higher in outer groves than inner groves. Similarly, mean percentage of flush shoots infested throughout the study period was 1.4-fold higher in outer groves than inner ones.

**Fig 3:**
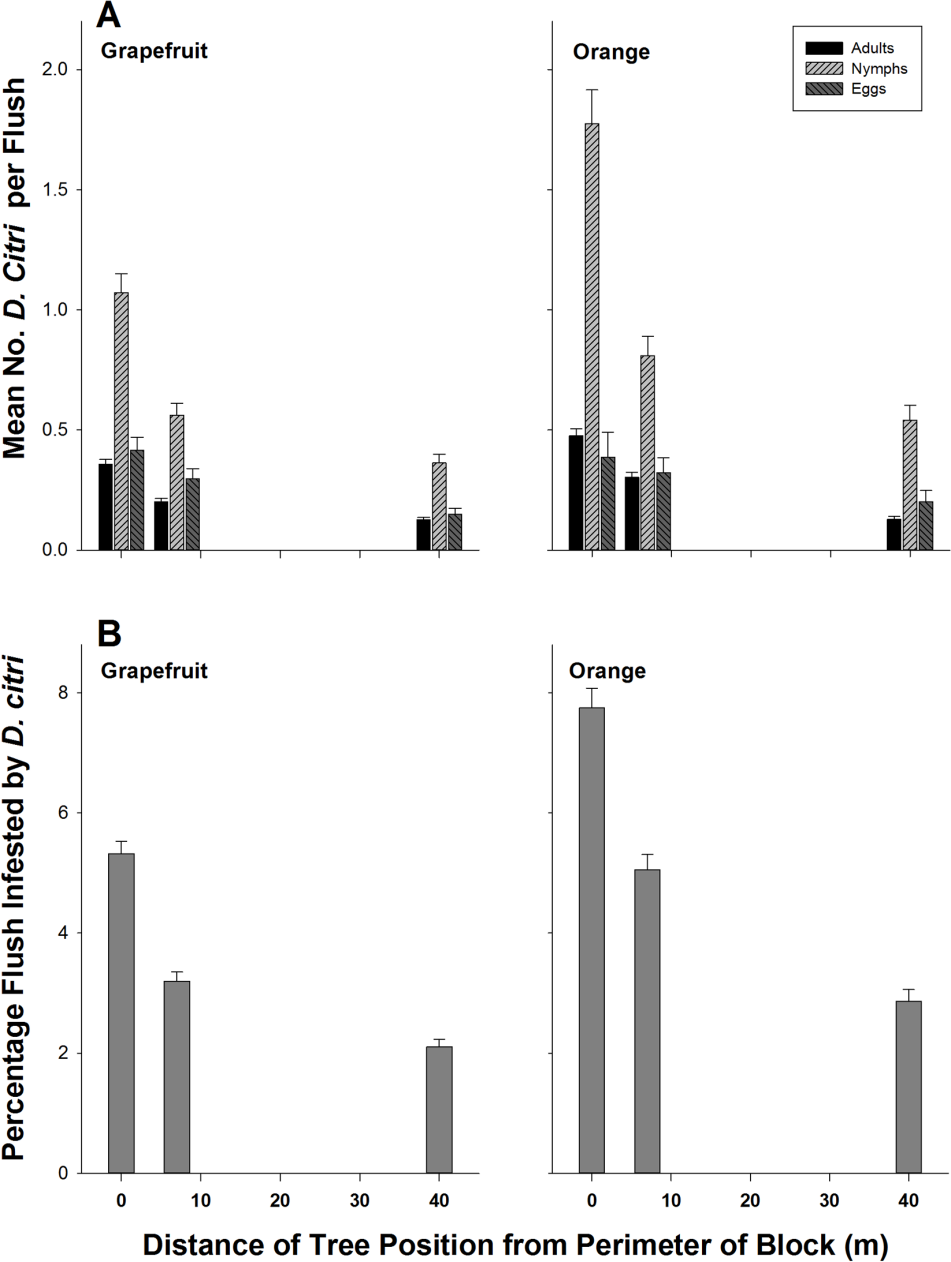
Mean number of *D*. *citri* life stages per flush shoot and percent flush infestation level in relation to tree location in grapefruit and sweet orange groves during area-wide surveys (2009–2010).

Independent of host plant species and grove location in the survey area, *D*. *citri* densities for egg, nymph and adult (*F* > 14.73, 99.45, 179.72; df = 2, 35,000; *P* < 0.0001) and flush shoot infestation level (*F* = 221.72; df = 2, 35,000; *P* < 0.0001) varied with tree position in the grove (Table 1). Flush shoot infestation levels and *D*. *citri* densities in citrus trees gradually declined from the edge of the grove to the interior. Least square mean discrimination using Tukey’s test showed three distinct groups for each *D*. *citri* variable recorded. The highest infestation levels and densities of *D*. *citri* were recorded on perimeter trees followed by adjacent trees, while interior trees harbored the least numbers of *D*. *citri* (Fig 3). The rate of decline in *D*. *citri* densities and flush infestation levels from the edge to the interior of the grove was similar for grapefruit and sweet orange, and was well described by an exponential decay function (*y* = a*exp [*-bx*], where *x* is the distance of tree position in meter from the edge of the grove and y the density of *D*. *citri* life stage) (Table 2). Based on these equations, *D*. *citri* flush shoot infestation level fell to one half of that recorded on perimeter trees within 33 m and 29 m of the grove edge in grapefruit and sweet orange, respectively. With the exception of the number of eggs on sweet orange, all *D*. *citri* developmental stages on interior trees decreased to 50% of their densities recorded on perimeter trees within 22 to 29 m of the grove edge (Table 2). Simple regression analysis between *D*. *citri* adult densities at different tree positions revealed good fits of models for both grapefruit and sweet orange (See S1 Appendix). About 90% and 76% of total variation in adult *D*. *citri* densities found on adjacent trees were explained by the number of adult psyllids recorded on perimeter trees for grapefruit and sweet orange, respectively. Similarly, 60–82% of the total variation in psyllid densities on interior trees was explained by psyllid densities on adjacent trees or perimeter trees. The slope of the regression lines indicated that each increase of 1 adult psyllid on perimeter trees resulted on only 0.4–0.5 and ∼0.3 psyllid increases on adjacent and interior trees, respectively (See S1 Appendix).

**Table 2:** Exponential decay function (*y* = a*exp [*-bx*]) describing the decrease in *D*. *citri* densities and infestation levels from the edge to the interior of the grove in area-wide surveys.

Host Plant	Parameter	Value	SE	R^2^	Distance to 50% reduction (m)
*D*. *citri* eggs					
Grapefruit	*a*	0.39	0.035	0.97	
	*b*	0.025	0.005		27.7
Orange	*a*	0.38	0.015	0.99	
	*b*	0.016	0.002		44.0
*D*. *citri* nymphs					
Grapefruit	*a*	0.86	0.233	0.73	
	*b*	0.025	0.017		28.3
Orange	*a*	1.33	0.463	0.65	
	*b*	0.027	0.002		25.9
*D*. *citri* adults					
Grapefruit	*a*	0.30	0.069	0.79	
	*b*	0.024	0.014		29.3
Orange	*a*	0.43	0.055	0.95	
	*b*	0.032	0.009		21.7
% flush shoots infested by *D*. *citri*					
Grapefruit	*a*	4.51	0.093	0.80	
	*b*	0.021	0.013		33.1
Orange	*a*	6.89	1.03	0.90	
	*b*	0.024	0.0089		29.5

### Initiation of *D*. *citri* infestation and spatio-temporal distribution in the field study

**Fig 4:**
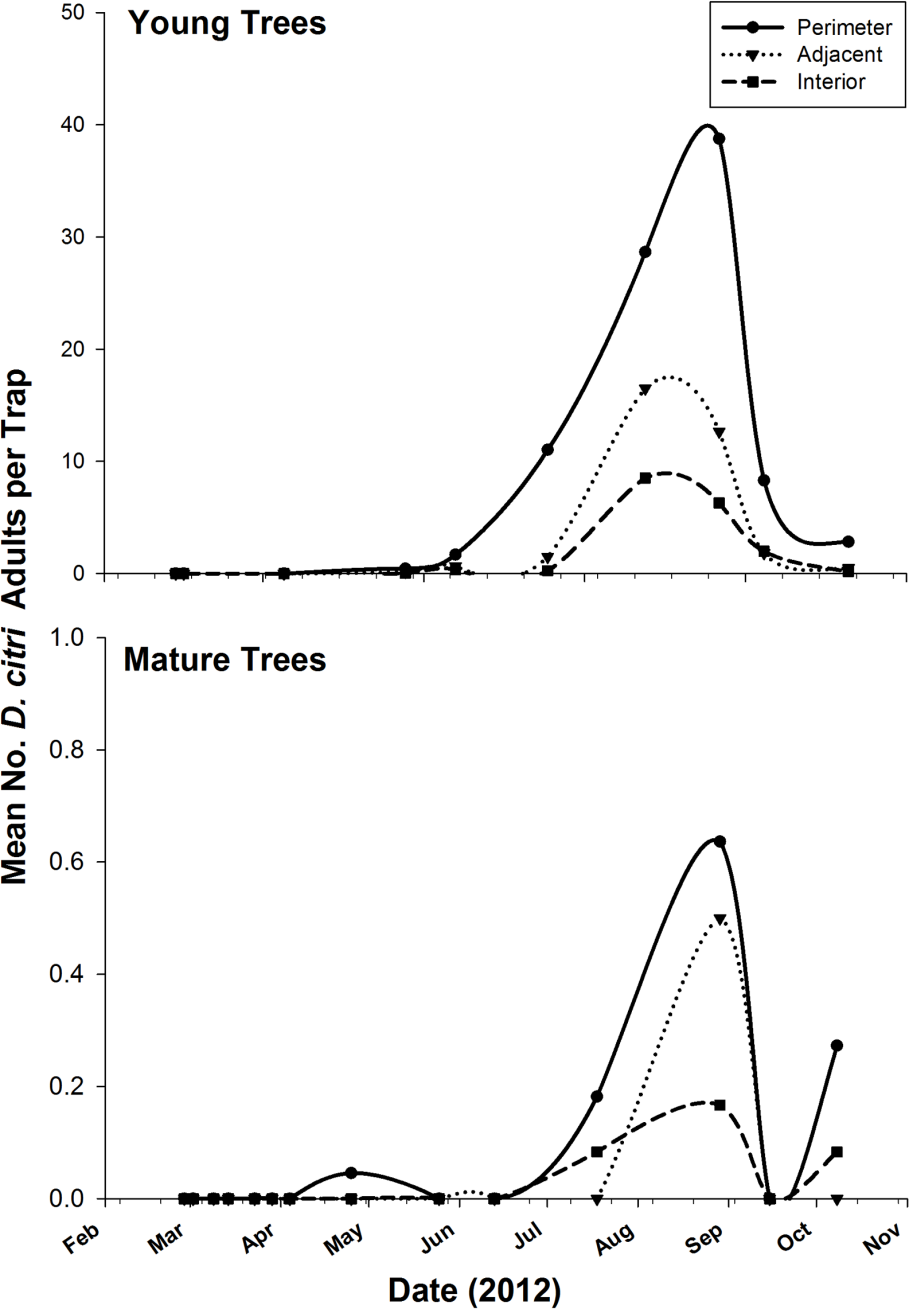
Mean number of *D*. *citri* adults caught on ACP-traps relative to tree position mature and young grapefruit groves in a field study (TAMUK-Citrus Center, 2012).

After two dormant sprays within a three-week period in January 2012, no adult psyllid was recovered from the two groves for three consecutive months (Fig 4). The first *D*. *citri* adults were caught on sticky traps in May and this initial *D*. *citri* detection on traps were made on the perimeter of the groves. Detection on *D*. *citri* adults on traps placed on adjacent and interior trees did not occur until 1 month after the initial detection on perimeter trees. Significantly more *D*. *citri* adults were caught on traps in the young grove compared to the mature grove (Fig 4). However, at all sampling dates, *D*. *citri* densities remained higher on perimeter than trees inside the grove (Fig 4). With the exception of one sampling date at the beginning of *D*. *citri* detection on interior trees in the mature grove, adjacent trees always harbored more psyllids than interior trees.

Cumulative numbers of *D*. *citri* trapped increased in a non-linear, sigmoidal pattern with time at all trapping positions in both the mature and young groves in field trials, except for traps placed on adjacent trees in the mature grapefruit grove (Table 3). Similarly, sigmoidal increases in the cumulative number of *D*. *citri* adults were observed in area-wide surveys for both grapefruit and sweet orange groves (Table 3). This sigmoidal relationship accurately described the temporal dynamics in cumulative *D*. *citri* numbers with *R* ^2^-values > 0.95 at all sampling transects in the groves. There are, however, significant differences in the rates of increase in *D*. *citri* numbers for each sampling position, with perimeter trees accumulating more adult psyllids than any other sampling location in the grove as shown by the parameter values of these sigmoidal functions (Table 3). In field trials, perimeter trees in both groves accumulated 3-fold more *D*. *citri* than adjacent trees while in the young grove these perimeters harbored 5-fold more adult psyllids than interior trees, as depicted by the maximum asymptote (*a*) of the sigmoid curves (Table 3). The inflection point–time to half-maximum of *D*. *citri* population accumulation (*x*_*0*_ in Table 3)–is between 220 and 234 Julian date or July 18-August 24, 2012, suggesting that half of adult psyllid population in both citrus groves accumulates during the last flush cycle of the year in September in Texas. In area-wide surveys, the rate of *D*. *citri* accumulation on perimeter trees was 1.6–1.8 and 2.7–3.5 folds higher than on adjacent and interior trees, respectively, and adjacent trees accumulated 16–2.2 times more adult psyllids than interior trees in both grapefruit and sweet oranges groves (See S1 Appendix).

**Table 3:** Parameter estimates and fit statistics of cumulative number of *D*. *citri* caught on ACP-trap during a growing season in young and mature grapefruit groves.

Sampling site	Parameter	Estimate	SE	t-value	*P*-value	*R* ^2^
Area-wide surveys						
Grapefruit groves						
Perimeter	*a*	8.95	0.39	22.81	<0.0001	0.98
	*b*	3.41	0.34	10.07	<0.0001	
	*x*_*0*_	13.42	0.48	27.78	<0.0001	
Adjacent	*a*	5.06	0.24	21.34	<0.0001	0.98
	*b*	4.05	0.36	11.11	<0.0001	
	*x*_*0*_	13.35	0.56	23.84	<0.0001	
Interior	*a*	3.26	0.23	13.99	<0.0001	0.97
	*b*	4.74	0.56	8.46	<0.0001	
	*x*_*0*_	13.45	0.93	14.42	<0.0001	
Sweet orange groves						
Perimeter	*a*	12.51	0.37	33.50	<0.0001	0.99
	*b*	3.88	0.24	16.50	<0.0001	
	*x*_*0*_	13.24	0.35	37.60	<0.0001	
Adjacent	*a*	7.81	0.36	21.68	<0.0001	0.98
	*b*	4.54	0.38	12.04	<0.0001	
	*x*_*0*_	13.01	0.60	21.86	<0.0001	
Interior	*a*	3.55	0.30	11.84	<0.0001	0.96
	*b*	5.04	0.63	7.97	<0.0001	
	*x*_*0*_	219.8	1.13	12.41	<0.0001	
Field trials						
Young grapefruit grove						
Perimeter	*a*	107.4	13.76	7.80	<0.0001	0.96
	*b*	32.1	7.45	4.32	0.0006	
	*x*_*0*_	234.0	11.23	21.0	<0.0001	
Adjacent	*a*	37.3	3.77	9.90	<0.0001	0.96
	*b*	30.0	6.48	4.63	<0.0001	
	*x*_*0*_	223.2	9.53	23.31	<0.0001	
Interior	*a*	19.8	2.17	9.15	<0.0001	0.97
	*b*	29.9	6.75	4.42	<0.0001	
	*x*_*0*_	229.0	9.71	23.59	<0.0001	
Mature grapefruit grove						
Perimeter	*a*	1.19	0.06	18.47	<0.0001	0.99
	*b*	22.7	3.61	6.28	<0.0001	
	*x*_*0*_	221.1	4.78	46.21	<0.0001	
Adjacent*	*-*	-	-	-	-	-
Interior	*a*	0.35	0.02	16.39	<0.0001	0.98
	*b*	25.3	4.09	6.18	<0.0001	
	*x*_*0*_	219.8	5.63	39.04	<0.0001	

* Parameters could not be estimated using a sigmoid function.

Positive and significant relationships were obtained between numbers of *D*. *citri* adult trapped at the different sampling positions for the young grove (See S1 Appendix). About 84–87% of the total variation of *D*. *citri* adults caught on traps placed on adjacent and interior trees were explained by trap catches on perimeter trees, while 98% of the variation in *D*. *citri* numbers recorded on interior trees was explained by trap catches on adjacent trees. Each increase of 1 adult psyllid on perimeter trees resulted in 0.4 and 0.03 more psyllids on adjacent and interior trees, respectively (See S1 Appendix), while the slope of the regression line indicated each increase in 1 psyllid on adjacent trees resulted in 0.51 increases of psyllid population on interior trees. No attempt was made to establish these relationships in the mature grove due to the very low numbers of psyllids caught on traps.

### Spatial niche occupation of *D*. *citri* in citrus groves–Area-wide surveys and field study

A total of 62 grapefruit groves with an average size of 3.9 ha and 34 sweet orange groves with an average size of 2.5 ha were sampled during the area-wide surveys. The proportion of trees present at each sampling location in the grove varied with the size of the grove, but on average 11.7%, 11.0% and 77.3% of trees for grapefruit and 12.6%, 11.8% and 75.6% for sweet orange were present on the perimeter, adjacent, and interior sampling locations, respectively. Independent of tree position in the grove, all *D*. *citri* developmental stages were recorded infesting flush shoots, but due to the fact that only adults select for host plants, preference indices were only calculated for this life stage. *Diaphorina citri* niche occupation preference significantly varied with tree location in the grove for both citrus cultivars. In addition, similar plant selection indices of trees were obtained for each of the three tree locations for both grapefruit and sweet orange (Table 4). *D*. *citri* preferentially selected perimeter trees for feeding and reproduction than adjacent and interior trees. Host plant selection preference of *D*. *citri* gradually declined from the edge to the interior of the grove. Perimeter trees were 1.5–1.7-, and 20-22-fold more likely to be colonized by adult psyllids than adjacent and interior trees, respectively in citrus groves. Using the standardized selection index (*B*_*i*_), *D*. *citri* exhibited stronger preference for perimeter trees, while interior trees were seemingly avoided in the grove (Table 4). No preference or avoidance was shown by adult psyllids toward adjacent trees.

**Table 4:** Spatial niche selection indices for *D*. *citri* adults in three habitat types in commercial citrus groves in South Texas (2009–2012). Habitat types are represented by perimeter, adjacent and interior trees.

Habitat	Proportion of trees available (No. of trees)	Mean No. of *D*. *citri*	Selection index *W*_*i*_ (Confidence limits)^1^	Standardized selection index (*B*_*i*_)^2^
Grapefruit in area-wide surveys (2009–2010)				
Perimeter	0.117 (146)	0.36	6.38 (2.71–10.1) a	0.608
Adjacent	0.110 (138)	0.30	3.80 (0.26–7.34) ab	0.358
Interior	0.773 (966)	0.13	0.22 (0.0–0.49) b	0.034
			χ2 = 34.25, *P* < 0.0001	
Sweet orange in area-wide surveys (2009–2010)				
Perimeter	0.104 (156)	0.48	6.58 (3.44–9.71) a	0.585
Adjacent	0.097 (148)	0.30	4.19 (1.10–7.30) a	0.389
Interior	0.797 (1196)	0.13	0.16 (0.0–0.40) b	0.026
			χ2 = 53.43, *P* < 0.0001	
Young grapefruit grove in field experiments (2012)				
Perimeter	0.19 (102)	9.17	4.31 (4.23–4.40) a	0.893
Adjacent	0.18 (99)	3.38	0.31 (0.26–0.36) b	0.064
Interior	0.63 (341)	1.77	0.21 (0.19–0.23) c	0.043
			χ2 = 158.0, *P* < 0.0001	
Mature grapefruit grove in field experiments (2012)				
Perimeter	0.31 (72)	0.08	2.52 (1.95–3.08) a	0.796
Adjacent	0.27 (62)	0.04	0.35 (0.0–0.81) b	0.111
Interior	0.42 (98)	0.02	0.30 (0.0–0.63) b	0.094
			χ2 = 158.0, *P* < 0.0001	

^1^ *W*_i_ = selection index of *D*. *citri*; Selection indices followed by the same letter are not significantly different at P = 0.05 (log-likelihood ratio [G] test).

^2^ *B*_*i*_ = standardized selection index for *D*. *citri*; standardized selection indices of (1/number of habitats), or 0.33 in this case, indicate no preference. Values above 0.33 indicate relative preference, and values below 0.33 indicate relative avoidance.

Similarly to area-wide survey data, *D*. *citri* exhibited a strong relative preference for perimeter trees in young and mature groves in the field studies, while both adjacent and interior trees were not preferentially colonized by adult psyllids (Table 4). Highest tree selection indices were obtained for perimeter trees, but no significant differences were obtained between the forage ratios of adjacent and interior trees for the mature grove.

### Visualization of *D*. *citri* counts in the field study

**Fig 5:**
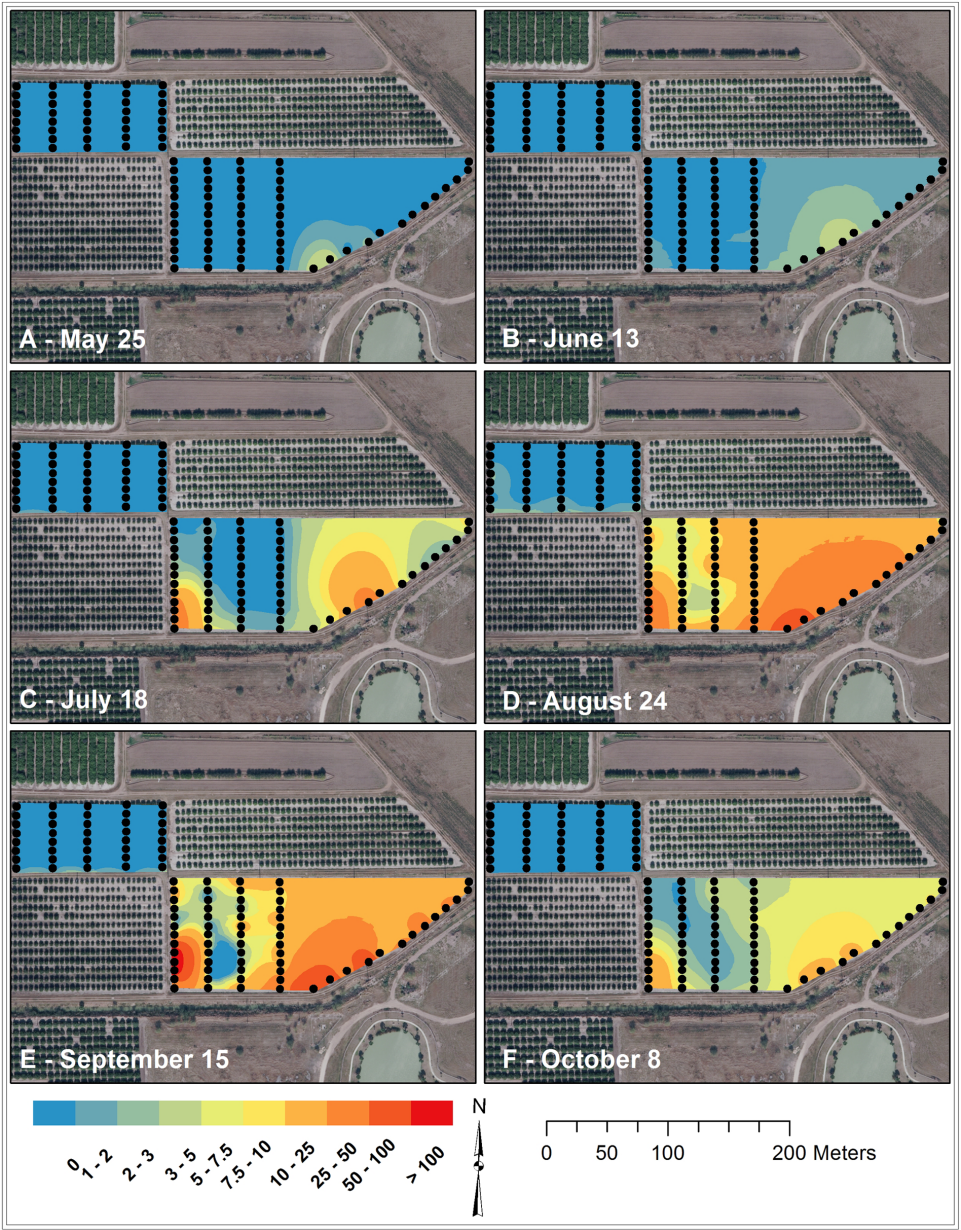
Contour maps of *D*. *citri* adults caught on ACP-traps deployed at different positions in young and mature grapefruit groves in a field study (TAMUK-Citrus Center, 2012).

The contour maps of *D*. *citri* adults trapped based on EBK clearly showed the preference for young citrus over mature plants and for perimeter trees (Fig 5). *D*. *citri* detection began on the southeastern edge of the young citrus grove and numbers increased to peak levels in September in the young grove. *D*. *citri* adults were detected on interior trees, but at lower numbers than on the perimeter trees. In the mature citrus, all the *D*. *citri* adults were captured on the perimeter traps, with the exception of one individual on an interior trap in August (Fig 4). By October, numbers of adults caught on traps were receding back to the perimeter (Figs 4 and 5).

## Discussion

Area-wide surveys and the detailed field study of *D*. *citri* population on citrus has led to a better understanding of its spatial niche occupation in groves, habitat preference, and field colonization strategies. In both area-wide surveys and the field study *D*. *citri* preferentially colonized perimeter trees in the groves and consequently flush shoot infestation levels and densities of all developmental stages were always highest on perimeter trees and gradually declined from the edge to the interior of the grove. This strong edge effect in *D*. *citri* spatial distribution in citrus groves may be due to several factors including citrus host plant characteristics and/or *D*. *citri* innate behavior. In commercial groves, mature citrus trees are large evergreen with dense canopy and perimeter or edge trees are also the first to be encountered by immigrating adults into the grove, and can therefore become a barrier for their arrestment. Once settled on these first encountered edge trees, adult psyllids will likely initiate oviposition when trees are flushing as evidenced by the larger numbers of eggs and nymphs on these perimeter trees, thus explaining the highest densities of all life stages observed on those trees relative to trees in other locations in the grove. The gradual decline in *D*. *citri* flush shoot infestation levels and densities from the edge to the interior of the grove may be a result of cascading within-grove dispersal of *D*. *citri*. Because *D*. *citri* adults move frequently between habitats[8], it is likely that secondary tree to tree colonization within the grove occurs from adult psyllids present on trees close to the edge of the groves. The significant and positive relationships obtained between numbers of adult psyllid on perimeter trees and adjacent or interior trees (See S1 Appendix) with explained variances over 90%, clearly indicated that *D*. *citri* numbers on adjacent and interior trees were dependent on numbers present on perimeter trees.

However, *D*. *citri* field niche occupation pattern in mature groves was similar to that observed in the young grove where trees did not reach their full size to constitute a close canopy that acts as screen to immigrating adults. Such observation may indicate that *D*. *citri* preference for border trees is an innate behavior, and not only dependent on tree size. *D*. *citri* is known to frequently change habitats [8] and preferentially living on border trees of the groves will be more conducive to locating suitable habitats. Adult psyllid preferentially select young expanding flush shoots for feeding, as these shoots are the only to support egg laying and immature development [4,24]. Edge trees are also characterized by higher illumination and we observed that they tend to produce flush shoots before inner trees in a grove, which can result in earlier *D*. *citri* colonization and consequently higher densities. The edge effects in *D*. *citri* colonization of citrus groves observed in the present study may explain the higher incidence of HLB-infected trees at the edge of citrus groves [9,10] due to the presence of higher vector densities and associated risk of inoculum transmission by infective *D*. *citri*. Similar edge effects have been observed in the spatial distribution of the Japanese beetle, *Popillia japonica* Newman (Coleoptera: Scarabaeidae) with higher beetle densities observed on edges of soybean fields than field interior, and specifically downwind hedges harbored more beetles than the other edges [25].

Numbers of *D*. *citri* nymphs and adults significantly varied with host plant cultivar. The higher densities of *D*. *citri* nymphs and adults on sweet orange than grapefruit indicates that host plant characteristics play an important role in *D*. *citri* host plant selection and/or immature survival. Patt and Sétamou [26] reported that *D*. *citri* exhibited differential attraction to flush shoots of its host plants. Higher numbers of adult *D citri* and flush shoot infestation levels in sweet orange than grapefruit may be the result of higher attraction and colonization of sweet orange groves by immigrating adults. Although no significant differences were observed between number of *D*. *citri* eggs per flush shoot between grapefruit and sweet orange, higher number of nymphs and adults were recorded on the latter cultivar. The comparative number of eggs recorded per flush shoot on both grapefruit and sweet orange suggests that the egg laying behavior of *D*. *citri* females is independent of host cultivar once the flush shoot has been selected, or that flush shoots of both citrus cultivars have comparative carrying capacity of *D*. *citri* eggs. However, the differential number of *D*. *citri* nymphs and adults recorded on flush shoots during the surveys showed that *D*. *citri* immature survivorship and adult emergence may be higher on sweet orange, suggesting that sweet orange is a more suitable host plant than grapefruit for *D*. *citri* development. Alves et al. [27] reported higher *D*. *citri* egg survivorship on sweet orange (*C*. *sinensis* [var. ‘Valencia’]) and orange jasmine (*Murraya paniculata*) than any other citrus host plants. Although no data on intensity of flush shoot production and flush age distribution were recorded in the present study, the host plant dependence of nymphal survival and adult emergence and/or flush shoot colonization, clearly demonstrates that flush shoot characteristics are important factors contributing to *D*. *citri* population fluctuations in citrus groves.

In addition to flush shoot characteristics, densities of all *D*. *citri* developmental stages were dependent on flush cycles in both citrus cultivars (Fig 1). New flush shoots are the primary resources for *D*. *citri* oviposition and immature development [4,24], and it is therefore not surprising that availability of new flush shoots was intimately related to *D*. *citri* population fluctuations. An increase in adult *D*. *citri* densities was recorded with the onset of a new flush cycle and a decrease was observed when flush shoots mature and no new flush shoots are present in the grove. Flores et al. [28] reported similar fluctuation in *D*. *citri* adult population in relation to flush cycles in citrus groves. Such fluctuations in *D*. *citri* populations with tree phenology clearly indicate immigrating adult psyllids are attracted into the groves at the beginning of a flush cycle, while most surviving and newly-emerged adults emigrate from the grove at its completion as shown by the decreasing numbers of adults caught on traps at the end of a flush cycle. During the entire field study and area-wide surveys, numbers of *D*. *citri* caught on traps or recorded were greatest on perimeter trees. This suggests that as observed during immigration, emigrating adults may also aggregate on border trees of a grove before their flight to new habitats or groves, thus explaining the preferential niche occupation of perimeter trees and the border effect observed in *D*. *citri* field distribution.

*D*. *citri* populations and flush shoot infestation levels significantly varied with sampling time. Percent flush shoots infested and densities of *D*. *citri* eggs and nymphs recorded during the late June to August flush cycles were significantly lower than those observed during any other flush cycle despite the presence of an adult population comparable to spring (March-early June) flush cycles (Fig 1). Such significant reduction in numbers of *D*. *citri* immatures per flush shoot and percentage of flush shoots infested may be due to the high temperature prevailing in South Texas between late June and August. During this warm season in South Texas (June-August), 47% of the total daily time is spent in the hot zone with temperature ranging from 29.4 to 37.8°C (http://weatherspark.com/averages/31732/Weslaco-Texas-United-States), occurring almost exclusively during daytime, the most active period of *D*. *citri* [29]. This range of temperature is well above the optimum temperature of 29.6°C for *D*. *citri* oviposition [30] and of 24–28°C for development [24,31]. Hot summer temperature may restrict *D*. *citri* movement and/or reduce its egg laying potential. In summer, citrus flush shoots also tend to mature more rapidly due to high temperature, thus the window of opportunity for *D*. *citri* egg laying and/or duration of flush shoot suitability for immature development may be reduced.

The edge effect observed in *D*. *citri* spatial distribution at the grove level was also recorded at the landscape level with higher *D*. *citri* densities and flush shoot infestation levels in groves at the outer edge of the study area than inner groves surrounded by other citrus blocks (Fig 2, S2 Appendix). This suggests that *D*. *citri* responds positively to its habitat edges, and may be an edge species. Such preference of *D*. *citri* for edge niches (Figs 3 and 4) may be characteristic of an insect species that moves frequently, and presence on outer trees and groves may favor such behavior. Grove and landscape edges may fundamentally be different from the interior due to the impact of both abiotic and biotic factors. Climatic factors such as wind speed, light intensity, and temperature regimes and differential soil fertility may alter the microclimate and environment of grove and landscape edges that can directly affect citrus tree phenology, *D*. *citri* biology and population dynamics, and/or the densities of other arthropod species interacting with *D*. *citri*. *D*. *citri* is mostly a diurnal insect and exhibits a strong phototropism [29], thus explaining its preferential colonization of border trees in a grove with higher illumination. Courtney and Courtney [32] reported higher butterfly oviposition rates on host plants along patch edges because of higher light levels and greater nutrient availability on these plants.

In addition to higher densities of *D*. *citri* on border trees, the detailed field study indicated that field colonization starts from the edge and progresses to the interior of the grove (Figs 4 and 5, S3 Appendix). Higher rate of *D*. *citri* population accumulation was also observed on those border trees than adjacent and interior trees in the grove throughout the active growing season, before a subsequent decline was recorded after the last flush cycle of the year (S4 Appendix). Such accumulation of *D*. *citri* on edge trees can be a result of inter-generational population growth during new flush cycles, the result of migration processes, or differential psyllid performance on border and interior trees. Due to the differential response of arthropod to edges [12,33], it may also be possible that *D*. *citri* natural enemies are more abundant in the interior of the groves, thus leading to higher psyllid mortality on trees inside the grove relative to perimeter ones. However, there was a density dependent relationship between inner tree colonization and densities on border trees (Tables 2 and 3). In addition to differential colonization preference of trees, studies may be warranted to evaluate the effects of tree nutritional quality, microclimate, or natural enemies on *D*. *citri* densities at different spatial niche in the grove.

The strong edge effects in *D*. *citri* spatial niche occupation in citrus groves demonstrated in the present study have important management implications for this pest. Because grove colonization starts from the grove edge where higher densities occurred throughout the year, sampling to determine *D*. *citri* presence and densities should only concentrate on grove edges without the need of sampling the whole grove. Such sampling protocol will be cost effective and increase the chance of psyllid detection. However because of higher *D*. *citri* densities on border trees, those edge densities may not be representative of actual densities across the entire grove and such a sampling scheme will lead to an overestimation of actual psyllid population in the grove. But such overestimation is beneficial for management purposes as it will lead to timely application of pesticides by growers.

Another potential benefit for the edge effect in *D*. *citri* grove colonization and spatial distribution is that citrus grove edges can be used as ecological traps on which control tactics can focus more than inner trees. Because adult psyllids are detected first on the grove edges (Figs 3 and 4), early targeted pesticide application on edge trees at the onset of grove infestation would substantially reduce *D*. *citri* population densities and prevent its spread and establishment to inner trees. Such targeted and timely border sprays would reduce the risk of HLB while minimizing pesticide input in the grove. The width of the edge treatment may depend on the depth of the edge effect or the ecotone. In the detailed field study where groves were almost devoid of psyllid for three months, initial infestation was found on the first two trees (perimeter and adjacent) of the groves for one month before any detection inside the grove (Figs 3 and 4). Such observation suggests that an edge management width encompassing an ecotone of two trees around the grove (i.e. first two rows on each side of the grove and first two trees on each row-end) will significantly slow *D*. *citri* spread and population increase within the interior of the grove. To be effective, edge treatment needs to be implemented following whole grove sprays and using *D*. *citri* detection surveys. Future studies evaluating the effectiveness of edge treatment for psyllid control in commercial citrus grove would be necessary to make informed management decisions based on *D*. *citri* spatial distribution patterns.

## Supporting Information

S1 Appendix Simple regression analysis describing the relationships between *D*. *citri* adult densities on flush shoots of citrus trees at different positions in a grove for area-wide survey and between adults caught on traps placed at different positions in the field study. (PDF)

S2 Appendix Raw data of *D*. *citri* area-wide surveys in commercial citrus groves during the 2009–2010 cropping season. (XLSX)

S3 Appendix *D*. *citri* distribution in young and mature groves in field study (TAMUK-Citrus Center, 2012). (XLSX)

S4 Appendix Cumulative *D*. *citri* numbers derived from area-wide surveys in commercial citrus groves during the 2009–2010 cropping season and from a field study in 2012 at TAMUK-Citrus Center (Weslaco, Texas). (XLSX)

S1 Fig Sample tree location in groves during the area-wide surveys in commercial groves (26°22'8.93"N, 98°16'14.32"W) during the 2009–2010 cropping season. (TIF)

S2 Fig Sampling transects and trap placement for *D*. *citri* adults monitoring during the detail field study in young (26° 8'11.99"N, 97°56'46.42"W) and mature (26° 8'14.54"N, 97°56'52.80"W) grapefruit grove (TAMUK-Citrus Center, 2012). (TIF)

## References

[1] Halbert SE, Manjunath KL. Asian Citrus Psyllids (Sternorrhyncha: Psyllidae) and Greening Disease of Citrus: a Literature Review and Assessment of Risk in Florida. Florida Entomol. 2004;87: 330–353. 10.1653/0015-4040(2004)087[0330:ACPSPA]2.0.CO;2

[2] Grafton-Cardwell EE, Stelinski LL, Stansly PA. Biology and management of Asian citrus psyllid, vector of the huanglongbing pathogens. Annu Rev Entomol. 2013;58: 413–32. 10.1146/annurev-ento-120811-153542 23317046

[3] French J V, Kahlke CJ, da Graça J V. First Record of the Asian Citrus Psylla, Diaphorina citri Kuwayama (Homoptera : Psyllidae), in Texas. Distribution. 2001;53: 14–15.

[4] Sétamou M, Flores D, French JV, Hall DG. Dispersion patterns and sampling plans for Diaphorina citri (Hemiptera: Psyllidae) in citrus. J Econ Entomol. 2008;101: 1478–1487. 18767763 10.1603/0022-0493(2008)101[1478:DPASPF]2.0.CO;2

[5] Bové JM. Huanglongbing: a destructive, newly-emerging, century-old disease of citrus. J Plant Pathol. 2006;88: 427–453.

[6] Hall DG, Gottwald TR. Pest management practices aimed at curtailing citrus huanglongbing disease. Outlooks Pest Manag. 2011;22: 189–192. 10.1564/22aug11

[7] Thomas CFG, Parkinson L, Griffiths GJK, Garcia AF, Marshall EJP. Aggregation and temporal stability of carabid beetle distributions in field and hedgerow habitats. J Appl Ecol. 2001;38: 100–116.

[8] Boina DR, Meyer WL, Onagbola EO, Stelinski LL. Quantifying dispersal of Diaphorina citri (Hemiptera: Psyllidae) by immunomarking and potential impact of unmanaged groves on commercial citrus management. Environ Entomol. 2009;38: 1250–1258. 10.1603/022.038.0436 19689907

[9] Aubert B. Integrated activities for the control of huanglongbing-greening and its vector Diaphorina citri Kuwayama in Asia. Proceedings of the 4th Acia Pacific International Conference on Citriculture, Thailand. 1990. pp. 133–144.

[10] Gotwald TR, Irey M, Gast T. The Plantation Edge Effect of HLB – A Geostatistical Analysis. IRCHLB Proceedings. 2008. pp. 305–308. Available: www.plantmanagementnetwork.org.

[11] Workneh F, Henne DC, Childers AC, Paetzold L, Rush CM. Assessments of the Edge Effect in Intensity of Potato Zebra Chip Disease. Plant Dis. 2012;96: 943–947. 10.1094/PDIS-06-11-0480 30727211

[12] Olson D, Andow D. Patch edges and insect populations. Oecologia. 2008;155: 549–558. 10.1007/s00442-007-0933-6 18157553

[13] Drapela T, Frank T, Heer X, Moser D, Zaller JG. Landscape structure affects activity density, body size and fecundity of pardosa wolf spiders (Araneae: Lycosidae) in winter oilseed rape. Eur J Entomol. 2011;108: 609–614.

[14] Haynes KJ, Cronin JT. Interpatch movement and edge effects: The role of behavioral responses to the landscape matrix. Oikos. 2006;113: 43–54. 10.1111/j.0030-1299.2006.13977.x

[15] Murcia C. Edge effects in fragmented forests: implications for conservation. Tree. 1995;10: 58–62. 10.1016/S0169-5347(00)88977-6 21236953

[16] Ries L, Fletcher RJ, Battin J, Sisk TD. ECOLOGICAL RESPONSES TO HABITAT EDGES: Mechanisms, Models, and Variability Explained. Annual Review of Ecology, Evolution, and Systematics. 2004 10.1146/annurev.ecolsys.35.112202.130148

[17] Arredondo IMJ. Abundance and population dynamics of Asian citrus psyllid Diaphorina citri Kuwayama (Hemiptera:Psyllidae) as affected by flush shoots in different host plants. 2009.

[18] SAS. SAS Procedures Guide. Version 9. SAS Institute; 2008.

[19] Zar JH. Biostatistical analysis 4th ed. Upper Saddle River: Prentice Hall; 1999.

[20] Savage RE. The relation between the feeding of the herring off the east coast of England and the plankton of the surrounding waters. Fish Investig Minist Agric Food Fish Ser 2. 1931;12: 1–88.

[21] Manly BF, McDonald L, Thomas DL, McDonald TL, Erickson WP. Resource Selection by Animals: Statistical Design and Analysis for Field Studies. London: Chapman and Hall; 2004.

[22] Krebs CJ. Ecological Methology. 2nd ed. New York: Addison-Wesley Educations Publishers, Inc.; 1999.

[23] Nansen C, Phillips TW, Morton PK, Bonjour EL. Spatial Analysis of Pheromone-Baited Trap Captures from Controlled Releases of Male Indianmeal Moths. Environ Entomol. 2006;35: 516–523. 10.1603/0046-225X-35.2.516

[24] Hall DG, Richardson ML, Ammar ED, Halbert SE. Asian citrus psyllid, Diaphorina citri, vector of citrus huanglongbing disease. Entomol Exp Appl. 2013;146: 207–223. 10.1111/eea.12025

[25] Sara S a, McCallen EB, Switzer P V. The spatial distribution of the Japanese beetle, Popillia japonica, in soybean fields. J Insect Sci. 2013;13: 36 10.1673/031.013.3601 23895634 PMC3738102

[26] Patt JM, Sétamou M. Responses of the Asian citrus psyllid to volatiles emitted by the flushing shoots of its rutaceous host plants. Environ Entomol. 2010;39: 618–624. 10.1603/EN09216 20388295

[27] Alves GR, Diniz A, Parra J. Biology of the Huanglongbing Vector Diaphorina citri (Hemiptera: Liviidae) on Different Host Plants. J Econ Entomol. 2014;107: 691–696. 10.1603/EC13339 24772551

[28] Flores D, Hall DG, Jenkins DA, Setamou M. Abundance of Asian Citrus Psyllid on Yellow Sticky Traps in Florida, Puerto Rico, and Texas Citrus Groves. Southwestern Entomologist. 2009 pp. 1–11.

[29] Sétamou M, Sanchez A, Patt JM, Nelson SD, Jifon J, Louzada ES. Diurnal Patterns of Flight Activity and Effects of Light on Host Finding Behavior of the Asian Citrus Psyllid. J Insect Behav. 2012;25: 264–276.

[30] Hall DG, Wenninger EJ, Hentz MG. Temperature Studies with the Asian Citrus Psyllid, Diaphorina citri: Cold Hardiness and Temperature Thresholds for Oviposition. J Insect Sci. 2011;11: 83 10.1673/031.011.8301 21870969 PMC3281434

[31] Fung YC, Chen CN. Effects of Temperature and Host Plant on Population Parameters of the Citrus Psyllid (Diaphorina citri Kuwayama). Formos Entomol. 2006;26: 109–123.

[32] Courtney SP, Courtney S. The “edgeeffect” in butterfly oviposition: causality in Anthocharis cardarnines and related species. Ecol Entomol. 1982;7: 131–137. 10.1111/j.1365-2311.1982.tb00651.x

[33] Porensky LM, Young TP. Edge-Effect Interactions in Fragmented and Patchy Landscapes. Conserv Biol. 2013;27: 509–519. 10.1111/cobi.12042 23531018

