# Supplementary material for: Living on the Edges: Spatial Niche Occupation of Asian Citrus Psyllid, Diaphorina citri Kuwayama (Hemiptera: Liviidae), in Citrus Groves
Source: PLoS One. 2015 Jul 20;10(7):e0131917. doi: 10.1371/journal.pone.0131917 (PMC4507854; doi:10.1371/journal.pone.0131917)
Supplement: S1 Appendix — (PDF) [file pone.0131917.s001.pdf]

Appendix 3. Simple regression analysis describing the relationships between *D. citri* adult densities on flush shoots of citrus trees at different positions in a grove for area-survey and between adults caught on traps placed at different positions in the field study**.**

Relationship between adult *D. citri* densities/flush	Intercept (*a*)	SE (*a*)	Slope (*b*)	SE (*b*)	*F*	R^2
Grapefruit (area-wide surveys)						
Perimeter (*x*) *vs* Adjacent (*y*)	0.052	0.018	0.42	0.030	189.53**	0.90
Perimeter (*x*) *vs* Interior (*y*)	0.025	0.018	0.28	0.030	86.67**	0.80
Adjacent (*x*) *vs* Interior (*y*)	-0.005	0.019	0.65	0.065	98.73**	0.82
Sweet orange (area-wide surveys)						
Perimeter (*x*) *vs* Adjacent (*y*)	0.03	0.042	0.54	0.065	68.96**	0.76
Perimeter (*x*) *vs* Interior (*y*)	-0.004	0.029	0.26	0.045	34.98**	0.61
Adjacent (*x*) *vs* Interior (*y*)	0.0003	0.028	0.42	0.072	34.08**	0.61
Young grapefruit (field study)						
Perimeter (*x*) *vs* Adjacent (*y*)	-0.364	0.904	0.41	0.057	51.58**	0.87
Perimeter (*x*) *vs* Interior (*y*)	-0.122	0.509	0.032	0.045	41.30**	0.84
Adjacent (*x*) *vs* Interior (*y*)	0.047	0.155	0.51	0.023	473.02**	0.98

** = highly significant ($P < 0.01$)
