# Supplementary figures and images for: Living on the Edges: Spatial Niche Occupation of Asian Citrus Psyllid, Diaphorina citri Kuwayama (Hemiptera: Liviidae), in Citrus Groves
Source: PLoS One. 2015 Jul 20;10(7):e0131917. doi: 10.1371/journal.pone.0131917 (PMC4507854; doi:10.1371/journal.pone.0131917)

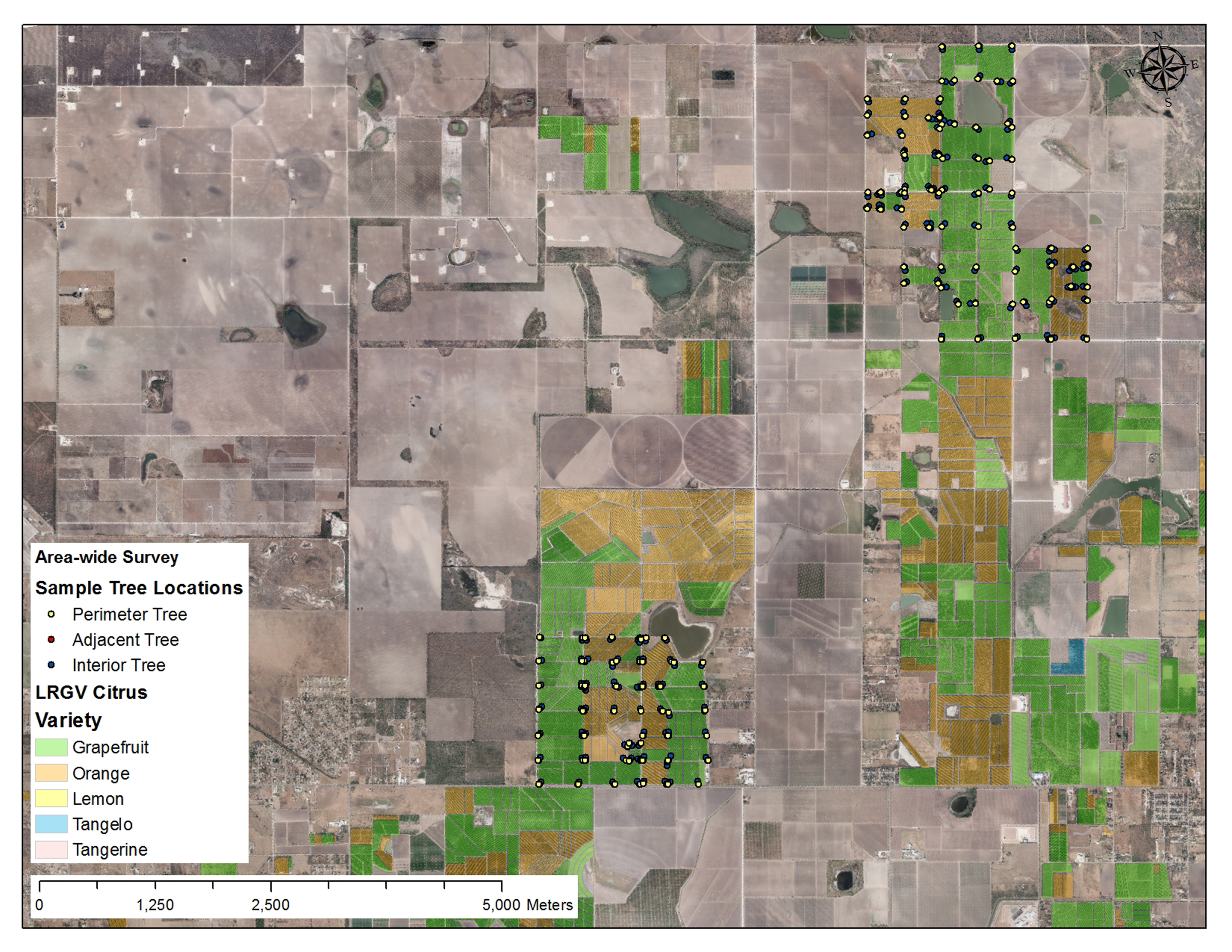

Supplement: S1 Fig — (TIF) [file pone.0131917.s005.tif]

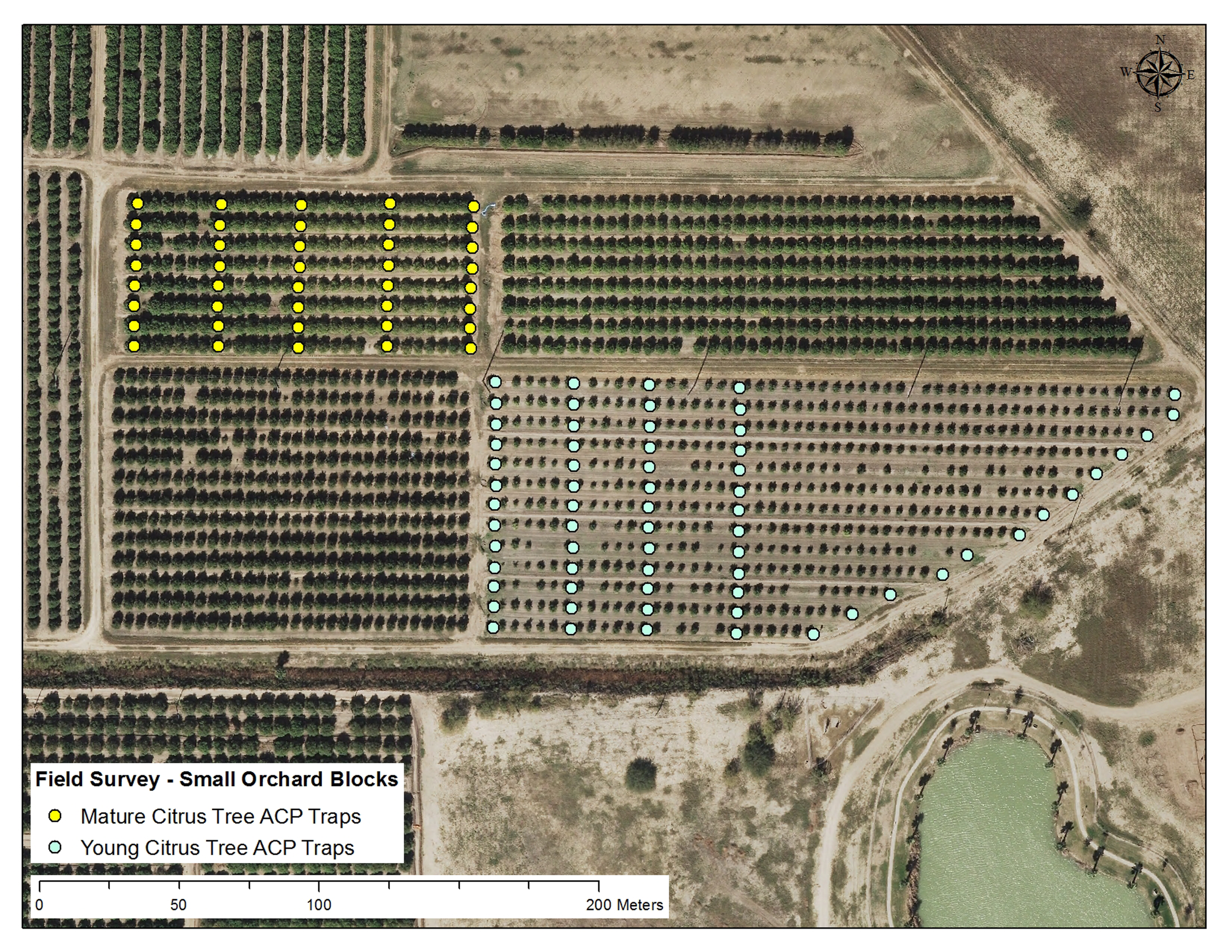

Supplement: S2 Fig — (TIF) [file pone.0131917.s006.tif]
